# An adaptive prediction and detection algorithm for multistream syndromic surveillance

**DOI:** 10.1186/1472-6947-5-33

**Published:** 2005-10-12

**Authors:** Amir-Homayoon Najmi, Steve F Magruder

**Affiliations:** 1 National Security Technology Department, The Johns Hopkins University Applied Physics Laboratory, Laurel, MD 20723-6099, USA

## Abstract

**Background:**

Surveillance of Over-the-Counter pharmaceutical (OTC) sales as a potential early indicator of developing public health conditions, in particular in cases of interest to biosurvellance, has been suggested in the literature. This paper is a continuation of a previous study in which we formulated the problem of estimating clinical data from OTC sales in terms of optimal LMS linear and Finite Impulse Response (FIR) filters. In this paper we extend our results to predict clinical data multiple steps ahead using OTC sales as well as the clinical data itself.

**Methods:**

The OTC data are grouped into a few categories and we predict the clinical data using a multichannel filter that encompasses all the past OTC categories as well as the past clinical data itself. The prediction is performed using FIR (Finite Impulse Response) filters and the recursive least squares method in order to adapt rapidly to nonstationary behaviour. In addition, we inject simulated events in both clinical and OTC data streams to evaluate the predictions by computing the Receiver Operating Characteristic curves of a threshold detector based on predicted outputs.

**Results:**

We present all prediction results showing the effectiveness of the combined filtering operation. In addition, we compute and present the performance of a detector using the prediction output.

**Conclusion:**

Multichannel adaptive FIR least squares filtering provides a viable method of predicting public health conditions, as represented by clinical data, from OTC sales, and/or the clinical data. The potential value to a biosurveillance system cannot, however, be determined without studying this approach in the presence of transient events (nonstationary events of relatively short duration and fast rise times). Our simulated events superimposed on actual OTC and clinical data allow us to provide an upper bound on that potential value under some restricted conditions. Based on our ROC curves we argue that a biosurveillance system can provide early warning of an impending clinical event using ancillary data streams (such as OTC) with established correlations with the clinical data, and a prediction method that can react to nonstationary events sufficiently fast. Whether OTC (or other data streams yet to be identified) provide the best source of predicting clinical data is still an open question. We present a framework and an example to show how to measure the effectiveness of predictions, and compute an upper bound on this performance for the Recursive Least Squares method when the following two conditions are met: (1) an event of sufficient strength exists in both data streams, without distortion, and (2) it occurs in the OTC (or other ancillary streams) earlier than in the clinical data.

## Background

Surveillance of Over-the-Counter pharmaceutical (OTC) sales as a potential early indicator of developing public health conditions, in particular in cases of interest to biosurvellance, has been suggested in the literature [1]. Sales of over-the-counter pharmaceuticals (OTCs) offer several advantages as possible early indicators of public health. They are very widely used [2], and reliable and detailed electronic records of their sales exist.

Another possible advantage is the timeliness of OTC sales relative to other observable events that might occur when the public health is threatened. This is a particularly difficult aspect since it requires the identification of specific events in all the data streams before a judgment can be reached as to the correlations and the timeliness of those events.

We have, in a previous article [3], provided evidence that when judiciously grouped, the OTC data show time-dependent correlations with clinical data, and that the present days values of the latter can be estimated well from the present and past values of the former using a set of linear filters *h*_*j*_[*m*], where the subscript *j *refers to the particular OTC product group (multiple groups are used) and the index *m *refers to the time step. If we denote the clinical data time series on day number *n *by *y*[*n*], and the OTC time series on the same day number *n *by *x*_*j*_[*n*], (the index *j *denotes the OTC product group), then the estimation problem discussed in our previous paper refers to using today's and past days' OTC data to estimate today's clinical data, in the sense that the estimated quantity is . The linear filters *h*_*j*_[*m*] are assumed to have a span of *M *points (days).

This estimate is to be compared with the actual value of the clinical data today. The "prediction" problem, the subject of the present paper, refers to an attempt to estimate future values of the clinical data using today's and past days' values of the OTC channels, i.e. the predicted quantity is now , *k *> 0. In the parlance of linear filter theory, the data set whose prediction is desired (the dependent variable) is termed the primary data channel. All other data sets (distinct from the primary channel) that are used to make the predictions are known as reference channels, otherwise known as independent variables. When the primary data set (the dependent variable) is used to predict its own values, then the primary channel is also the reference channel (the independent variable).

We present a prediction method based on an adaptive recursive least squares filter. In addition, we compare these predictions, which we term auto predictions, with similar predictions that use the same method applied to the clinical data alone without referencing any OTC channels. It is our contention that when the auto prediction results (i.e. when using the clinical data in the past to predict its future values) are equally (in the sense of minimum squared error) effective as or better than those predictions based solely on OTC streams, in all time intervals, then it is highly probable that no event of interest to biosurveillance actually exists in the clinical data. This is based on the fundamental premises of linear optimal predictors that a nonstationary and relatively short duration event superimposed on an otherwise stationary and predictable background cannot be predicted from the stationary background data alone. We argue that the best performance comparison, in the context of a biosurveillance system whose objective is to detect an outbreak early, among all method/data stream combinations is tied closely to the existence of such events. Lacking any real specific events of sufficient signal strength, we perform a study based on simulated events in order to compute an upper bound on the indicated performances. We emphasize that the system whose performance we are investigating here is a predict and detect system, in the sense that it uses historical clinical and other ancillary data streams in order to predict clinical data many days into the future. The detection performance is then based on a study of probabilities of true detections versus the probabilities of false alarms.

The meaning of an upper bound on the detection performance in this context is in the following sense. Given a data stream *y*_*t *_that includes an event of short duration then the detection performance of a specific prediction method, is related to the quantity  where  is the predicted value of the data stream when an event exists and *y*_*t *_is the value of the data stream in the absence of the event. This predicted value could be based on the data stream itself, or it could be based on a combination of the data stream and several other correlated data sets. In a real-time situation one might perform detections based on the quantity , where  is a prediction of the data stream in the absence of the signal, because the actual quantity *y*_*t *_is not available when the predictions are made at *t *- Δ*t*. We contend that an upper bound on detection performance is obtained when we use the "actual background" *y*_*t *_instead of the "predicted background" .

## Methods

### Data grouping and recursive least squares prediction

JHU/APL is currently collecting large quantities of daily OTC sales data. We receive sales records of 622 different products under the general category of cold remedies from a single vendor, with similar numbers from other vendors. Many of these products are used to treat very similar conditions. Product sales from some of these product groups are known to be good indicators of the corresponding clinical data. For instance, chest rub sales are highly correlated with the count of physician diagnosis of acute bronchitis or acute bronchiolitis [4].

The OTC products of interest were grouped based on a combination of the syndromes the product is intended to alleviate, the physical description of the product (e. g. a pill, a powder, a lip balm, etc.) and the age/sex group the product is targeted for. There were 15 syndrome groups, 15 physical types, and 4 target age/sex groups (mostly age, but 4 of the products were designated as intended especially for women). Some combinations contained no products, but there were a total of 92 combinations that did, so there was still some need for aggregation of these groups. The aggregation procedure has been reported elsewhere [5]. The groups we eventually used are shown in Table 1. The clinical data are counts of outpatient encounters, based on physicians diagnoses (according to the *International Classification of Diseases, Ninth Revision *(ICD9) standards [6]) reported in insurance claims that fall within a particular set of acute respiratory conditions (see table 1). Encounters were included only for patients 12 years old and older. The encounters are further restricted to include only patients living in the National Capitol Region (NCR), which includes the District of Columbia, Baltimore, suburban portions of Maryland and the Washington suburbs in Virginia. The encounters are time-tagged according to the day of occurrence.

**Table 1:** OTC Adult Medication Product Groups

**Product Group Name**
ALLERGY
BRONCHIAL
COLD-ALLERGY
COUGH
FLU
POWDER
SINUS
THROAT

Here, we consider the clinical data, the dependent variable, as the primary data channel (in the parlance of adaptive filter theory) whose values are to be predicted. The OTC product groups (the independent variables) are then used to predict the daily clinical data in the following manner. Today's and several past days' OTC data are combined to make a future clinical data prediction, which is then compared to the actual value of that day's clinical data when it becomes available, and the error is used to update the filter coefficients in such a way as to minimize the square of the error. For simplicity and to illustrate the method we consider the estimation problem in which there is only one reference channel whose value at each time *n *is denoted by *x*[*n*] (note that the subscript *j *denoting the particular product group is now missing since we are using an example with only 1 product group). The latter is used to estimate the present value *y*[*n*] of the primary channel (the dependent variable – office visit data). The estimation equations, once put into the recursive form are then easily generalized to the prediction problem. A linear estimate of the primary channel in terms of a single reference channel is given by , where we have assumed a filter of length *M*. The last equation can be written as a vector dot product , where *h*= [*h*[0], *h*[1],..., *h*[*M *-1]]^*T*^, *x*[*n*] = [*x*[*n*], *x*[*n *-1],..., *x*[*n *- (*M *- 1)]]^*T*^, and the superscript *T *denotes the transposition operation (the transpose of a row vector is a column vector). A linear predictor of the primary channel (the dependent variable) at *k *steps ahead is then given by .

Clearly we could perform a similar prediction process when we use the clinical data by itself instead of the OTC data streams, as well as simply including the clinical data as an "additional" reference data stream. Let *P *denote the number of days ahead to predict, *M *denote the number of linear filter coefficients, and *N *denote the number of OTC data channels. Then the filter vector *h*will have *M *× *N *elements when we predict the clinical data using the OTC channels, and it will have *M *elements when we use the clinical data to predict itself, and (*M *+ 1) × *N *elements when we combine the OTC channels and the clinical data to predict the clinical data. The covariance/correlation matrix of the reference channels will then be a square matrix of the appropriate dimension in each case. The filter application and updates are recursive. Denoting the clinical data (the dependent variable) on day *n *by *y*[*n*] and the reference data (the independent variables) by *x*_*j*_, the Recursive Least Squares *P *– *days *ahead prediction equation is [7] Where *L *denotes the number of reference data streams and it is equal to *N *when only OTC data are used, or 1 if the clinical data is used to predict itself, or *N *+ 1 if both clinical and OTC channels are used to predict the clinical data. This step is repeated as many times as required in order to obtain the predicted values . The recursion equations and the new method of minimum multiple look error feedback is described in Additional file 1. We should point out that when we use the clinical data alone (i.e. for self prediction) then the prediction equation is of the form , where the subscript *j *could be left out since only one filter is used.

**Figure 1:**
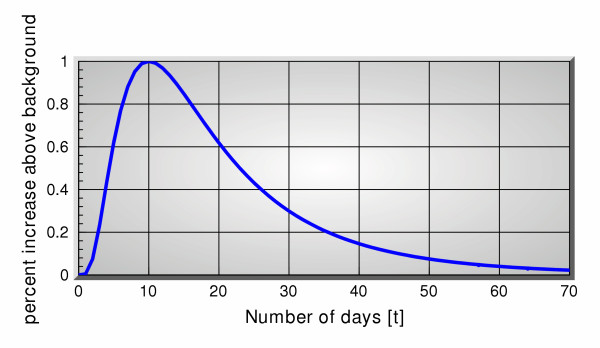
Simulated multiplicative event.

Our simulated signal was constructed by combining the following assumptions about an event of interest that can be reasonably expected to arise if a biological attack were to occur. The event is the result of a deterministic multiplicative signal *s*(*t*), in the sense that if *y*(*t*) denotes the clinical data in the absence of the signal, the presence of the signal will lead to the following clinical data *y*(*t*){1+*s*(*t*)}. The corresponding event has a sharp rise of about 5–10 days from a minimum of 0 to a maximum value that shows the corresponding percent increase of clinical data at the peak of the outbreak, above the normal "background" number. We consider the rise time of about a week to be a reasonable assumption based on observations of infectious disease characteristics [8]. In addition, we assume that the event has a fall off period of about twice that of the rise time. This signal can be easily modeled by a function of the form . The log-normal shape is based on observations as reported by Sartwell [8]. Figure 1 shows a specific example matching the requirements of an event described above.

The simulation consists of applying this deterministic signal with a given maximum value to the OTC data, on any given day, and applying the same deterministic signal to the clinical data with a time delay. Then we compute predictions of the clinical data once using OTC data only, and a second time using the clinical data itself. In both cases we use the predicted clinical data for detection and the detector output is , where  denotes the predicted value of the clinical data on day *t*. The predictions are performed once with no signal present (to compute false alarms), and once with signal present to compute true detections, and for all day numbers 100 through 550. A range of thresholds are used to calculate the total number of detections and the total number of false alarms. These numbers are then averaged over the total number of days to give the probability of detection *p*_*d *_and the probability of false alarm *p*_*fa*_, both of which are functions of threshold. The Receiver Operating Characteristic (ROC) curve is then obtained by eliminating the threshold variable and plotting *p*_*d *_as a function of *p*_*fa*_. This curve provides the most concise form of evaluating the performance of any detection system [9]. The best performance is, by definition, a horizontal line *p*_*d *_= 1, while the worst is the line *p*_*d *_= 0, for all values of *p*_*fa*_. The 45° line represents the performance of a hypothetical detector that decided on the presence of a true signal by tossing a fair coin, i.e. equal probabilities of detection vs false alarm.

Simulation parameters are as follows. The signal maximum strength takes on values 10%, 100% and 200% (percentage increases refer to the background counts). We have chosen 2 signal lag times of 5 and 10 days (lag times refer to the lag between the application of the maximum signal strength to the OTC channels and the office visit count channel). The predictor uses a filter length of 5 days and we try 2 sets of predictions: 5 days ahead and 10 days ahead.

## Results and discussion

**Figure 2:**
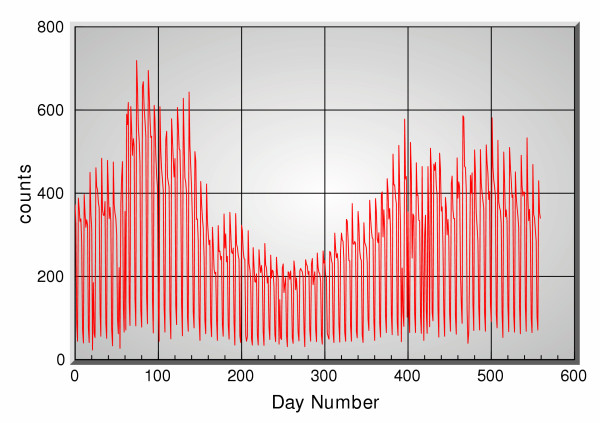
Office visit data [clinical data] between 11/1/2001 and 5/14/2003.

**Figure 3:**
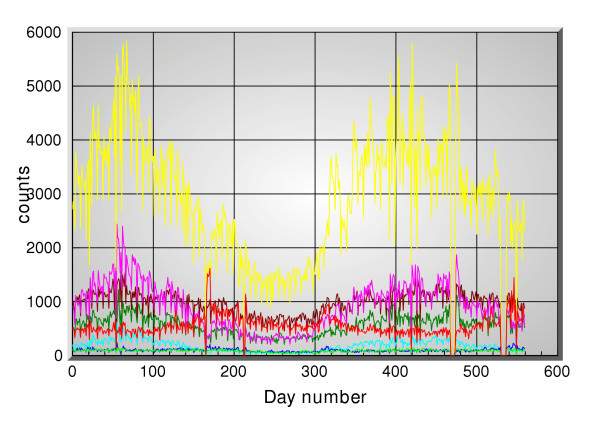
OTC sales data for the same period.

**Figure 4:**
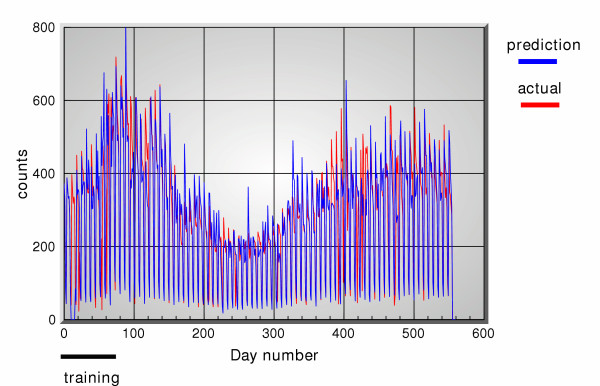
5-days ahead predictions of office visit data using OTC sales data, versus actual data.

**Figure 5:**
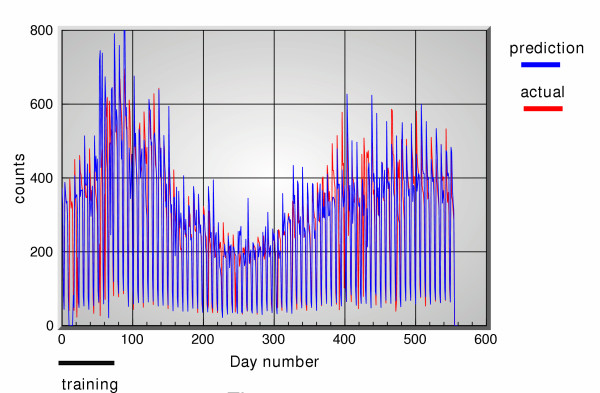
5-days ahead predictions of office visit data using OTC sales data and office visit data, versus actual data.

**Figure 6:**
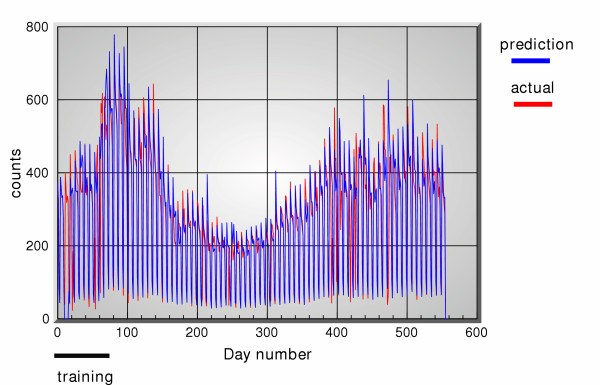
5-days ahead predictions of office visit data using OTC sales data and office visit data, versus actual data.

Figure 2 shows the actual clinical data, and figure 3 shows all the OTC channels, for the period 11/1/2001 through 5/14/2003 consisting of 560 days. Figure 4 shows the 5-days ahead prediction results using only the OTC channels. Figure 5 shows a similar output when the office visits data are added in as another reference channel. Figure 6 shows the output when only the office visits data are used to predict their own future values, i.e. OTC channels are not used here. All results use a 5 point filter, i.e. for each OTC product group *j *the corresponding filter is *h*_*j*_[*m*] and 0 ≤ *m *≤ 4. The effective memory of each filter is set at approximately 1 month.

**Figure 7:**
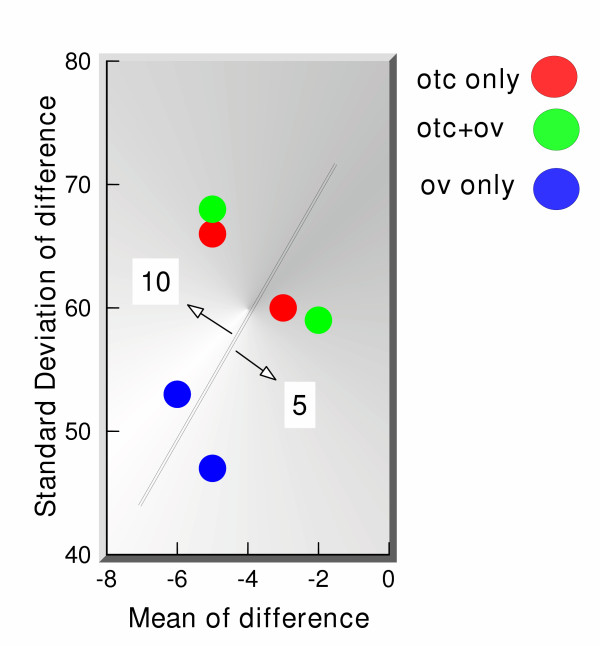
Performance characteristics of 5-days and 10-days ahead predictions, using OTC sales data alone, OTC data plus office visit data, and office visit data alone.

A simple measure of the effectiveness of the predictor performance (not the detector performance) is a plot of the mean versus standard deviation of the difference between the actual and the predicted values (prediction error vector). Figure 7 shows the means versus standard deviations for all 3 cases and for 5-days ahead as well as 10-days ahead predictions. The prediction error vector was computed between days 100 and 550, to allow for filter initialization in the beginning; we could have started making predictions as early as 50 days from the beginning since the effective memory of the filter is set at 30 days, but chose to begin making predictions at day 100 to be absolutely safe. These prediction results are quite encouraging and show significant correlations between OTC and office visit data, in the sense that the predictions are quite close to the actual values. What is perhaps more surprising, is the fact that using office visit data for self prediction apparently has the lowest error. Although these errors are computed over the entire time section, there are no time intervals over which the self prediction results are worse than those when the OTC are used to predict the clinical data.

Our interpretation is that this particular office visit data has sufficiently strong autocorrelations at long lags that allows for a better prediction when compared to the predictions made using the cross correlations. We emphasize that one cannot draw any conclusions as to the best combination of method/data for prediction from these results when in fact no identifiable and significant events of interest exist in the present data set. For instance, one cannot state that OTC data can be safely ignored in the prediction problem in favour of using the clinical data itself. In order to illustrate this point and to place an upper bound on detection performance of a biosurveillance system that relies on predicting the clinical data from OTC and/or clinical data, we have performed an analysis based on simulated events superimposed on the present data sets.

**Figure 8:**
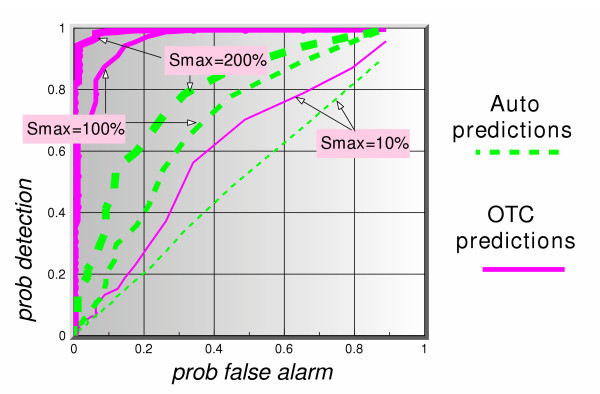
ROC curves of auto-predictions (office visit data alone to predict itself), and OTC predictions (OTC data to predict office visit data), for 3 signal strengths, using a simulated actual lag of 5 days between OTC and office visit data and a 5-days ahead predictor.

**Figure 9:**
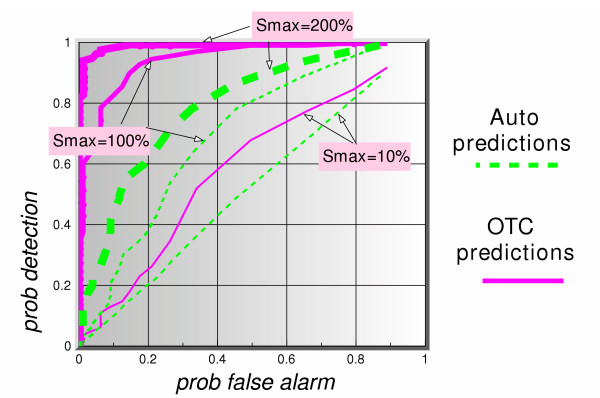
ROC curves of auto-predictions (office visit data alone to predict itself), and OTC predictions (OTC data to predict office visit data), for 3 signal strengths, using a simulated actual lag of 10 days between OTC and office visit data and a 5-days ahead predictor.

**Figure 10:**
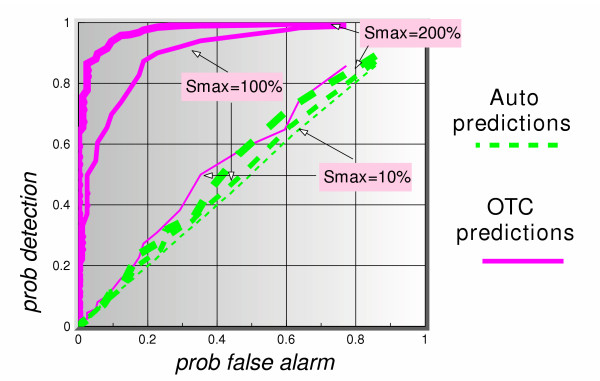
ROC curves of auto-predictions (office visit data alone to predict itself), and OTC predictions (OTC data to predict office visit data), for 3 signal strengths, using a simulated actual lag of 10 days between OTC and office visit data and a 10-days ahead predictor.

**Figure 11:**
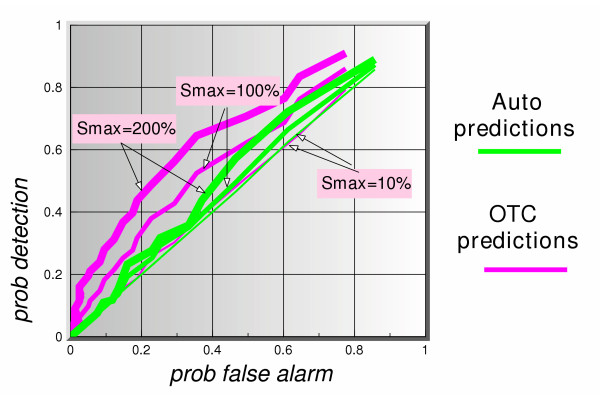
ROC curves of auto-predictions (office visit data alone to predict itself), and OTC predictions (OTC data to predict office visit data), for 3 signal strengths, using a simulated actual lag of 5 days between OTC and office visit data and a 10-days ahead predictor.

Figure 8 shows the ROC curves (*p*_*d *_vs *p*_*fa*_) for a 5-day lag and 5 days ahead prediction for all three signal maximum amplitudes. The dotted lines represent the auto-predictions made using only the clinical data. The solid lines show the predictions using the OTC data. The thickness of the lines in each case represents the signal maximum amplitude. Based on this figure alone, we can summarize these results as follows. Given the assumptions in this simulation, the auto-predictions do not appear to perform well in a predict-ahead and detect surveillance system. For instance, even at signal maximum amplitude of 200%, for a  = 0.2, corresponding to a false alarm rate of once every 5 days, the probability of detection when using auto-predictions is barely above 60%, whereas if one uses the OTC predictions that probability is 100%. If a signal maximum amplitude is lowered to 100%, the auto-prediction probability of detection is down to 40%, and that of OTC predictions is still a very healthy 97%. These results continue to hold so long as the lag value is larger than or equal to the number of prediction days, as can be seen clearly in figures 9 and 10. Figure 11 shows a dramatic fall in performance when the latter condition is not satisfied, i.e. when the number of prediction days exceeds the lag number. Clearly, if we try to predict "too many" days ahead (irrespective of the lag number), the detection results worsen considerably. The present data set predictions appeared to hold to about 12 days ahead.

Our choice of the synthetic signal requires further discussion. Since we are interested in placing upper bounds on the performance of a multi-stream syndromic surveillance system that uses a prediction method to detect an outbreak we decided to concentrate on a type of epidemic curve that has a reasonably fast rise time and a slower fall off. We chose the 1-week rise time and 2-weeks fall off because they are reasonable numbers in the context of early detection of most biological attack scenarios. It so happens that these numbers appear to fit the observations by Sartwell and so we used a log-normal shape. It turns out the results are quite insensitive to the analytic form of the signal, for instance, we could have used a "triangle" signal with the same rise-time characteristics and reached similar conclusions.

Finally we should discuss our results in view of the fact that we applied the same multiplicative signal in all data streams without distortion. A complete simulation study of the performance of a multi-stream syndromic surveillance system that uses a prediction method to detect an outbreak would include all possible signal distortions (including amplitude reduction, but also changes in the shape of the signal), and all reasonable time delays; this is a huge task and well beyond the scope of this publication. What we have attempted here is obtaining an upper bound on this performance by varying the time delay and the maximum amplitude of the signal but keeping the signal undistorted. Any distortion of the signal would clearly degrade the ROC curves. In the absence of a general theory of infectious disease evolution and the uncertainties associated with the impact of an infectious disease outbreak upon all the data streams in a multi-stream syndromic surveillance system we have found performance upper bounds on the limited number of cases we have studied, in conjunction with the prediction algorithm presented here.

## Conclusion

Based on our simulation results we can state the following broad conclusions regarding a multistream syndromic surveillance system that operates by predicting the clinical data several days in advance and issuing early warnings if the predicted values exceed a given thershold. This predict-and-detect system must include ancillary data streams (such as OTC) with established correlations with the clinical data, and a prediction method that can react to nonstationary events sufficiently fast. Any predictions of the clinical data using only the clinical data, i.e. relying on self-correlations of the clinical data rather than cross-correlations with other data streams such as OTC data, can be an effective estimate of the background conditions. Whether OTC (or other data streams yet to be identified) can provide the best source of predicting clinical data is still an open question. The system must also include a prediction algorithm that can react sufficiently fast to nonstationary changes. The Recursive Least Squares Minimum Distance Error algorithm presented here seems to satisfy this condition. Finally, we have no way of knowing the likelihood that events of interest will always be present in both the clinical data and the ancillary streams, without significant distortion, and with reasonable time lags. But if any event satisfies these conditions, we have provided the framework for a system that has an excellent chance of detecting it in advance.

## Abbreviations

OTC: over the counter (medications).

## Competing interests

The author(s) declare that they have no competing interests.

## Authors' contributions

The idea of predicting clinical data using OTC, and the algorithm using recursive least squares with feedback of minimum error among multiple looks, and the associated computer programmes were developed by A. H. Najmi. The OTC data were grouped from among a much larger set into a small set used in this study, via a maximum likelihood method developed by S. F. Magruder.

## Pre-publication history

The pre-publication history for this paper can be accessed here:



## Supplementary Material

Additional File 1 Recursive Least Squares Prediction with minimum distance multiple look error feedback. Click here for file
