# Supplementary material for: An adaptive prediction and detection algorithm for multistream syndromic surveillance
Source: BMC Med Inform Decis Mak. 2005 Oct 12;5:33. doi: 10.1186/1472-6947-5-33 (PMC1266371; doi:10.1186/1472-6947-5-33)
Supplement: Additional File 1 — Recursive Least Squares Prediction with minimum distance multiple look error feedback. [file 1472-6947-5-33-S1.pdf]

Additional file 1 - Appendix

Recursive Least Squares Prediction with minimum distance multiple look error feedback

Reference 7 describes the *estimation* problem, but the extension to the *pure prediction* problem is straightforward. Although our work involves multiple independent data streams (multiple reference channels), we limit this appendix to one independent data stream (reference channel), e.g. a single OTC product group, without loss of generality. Let $\hat{y}\left[n+k\right]$ denote the predicted time series (the dependent variable) when data at time steps $\leq n$ are available, i.e. we are making a prediction $k>0$ steps ahead $\hat{y}\left[n+k\right]=\sum_{m=0}^{M-1}h\left[m\right]x\left[n-m\right]$. We define a performance index $\varepsilon_n=\sum_{l=0}^{n}\lambda^{n-l}\left|e\left[l\right]\right|^2$ where the error at each step is defined by $e_l=y_l-\hat{y}_l$, and λ is the "forgetting factor", introduced to enforce adaptation of the filter to the most recent changes in the data. The role of the forgetting factor can be understood by considering the quantity n_λ where

$$n_\lambda=\frac{\sum_{n=0}^{\infty}n\lambda^n}{\sum_{n=0}^{\infty}\lambda^n}$$

. Now the expression on the right hand side is easily found to be

$$n_\lambda=\frac{\lambda}{1-\lambda}$$

which represents a measure of the <u>effective memory</u> of the filter. Smaller values for λ correspond to shorter memory lengths n_λ which will enable better tracking of nonstationarities. Defining the matrix element $R_{ij}\left[n\right]=\sum_{l=0}^{n}\lambda^{n-l}x\left[k-i\right]x\left[k-j\right]$, and the vector element $r_i\left[n\right]=\sum_{l=0}^{n}\lambda^{n-l}y\left[k\right]x\left[k-i\right]$, the Normal equations **[7]** at any step (day number n) are $\underline{\underline{R}}\left[n\right]\underline{h}^{(n)}=\underline{r}\left[n\right]$, where the dependence of the filter on n is explicitly shown by the superscript on the filter vector, which itself is a vector of length M denoting the span of the filter over M days. The quantities $\underline{\underline{R}}\left[n\right]$ and $\underline{r}\left[n\right]$ satisfy rank-one update equations which describe the way these quantities change from one day to the next: $\underline{\underline{R}}\left[n\right]=\lambda\underline{\underline{R}}\left[n-1\right]+\underline{x}\left[n\right]\underline{x}^T\left[n\right]$ and $\underline{r}\left[n\right]=\lambda\underline{r}\left[n-1\right]+\underline{x}\left[n\right]y_n$. Standard rank-one update techniques from matrix algebra then lead to the following recursive least squares algorithm (if we denote apriori quantities by a subscript 0, and apostiori quantities by a subscript 1):

Inverse covariance and Kalman gain: $\underline{\underline{P}}_0 = \underline{\underline{R}}_0^{-1}$ $\quad \underline{K}_0 = \underline{\underline{P}}_0 \underline{x}$

Likelihood variables: $\nu = \underline{K}_0^T \underline{x}$, $\quad \mu = (1+\nu)^{-1}$

Inverse covariance and Kalman gain: $\underline{K}_1 = \mu \underline{K}_0$ $\quad \underline{\underline{P}}_1 = \underline{\underline{P}}_0 - \underline{K}_1 \underline{K}_0^T$

Prediction and error: $\hat{y}_0 = \underline{h}_0^T \underline{x}$ $\quad e_0 = y - \hat{y}_0$

Filter update: $\underline{h}_1 = \underline{h}_0 + e_0 \underline{K}_1$

The recursion begins with a zero filter (i.e. a vector of length M and zero for every element) and a covariance matrix that is a large multiple of the unity matrix.

The multiple look feature introduced in this paper relates to the prediction error that is fed back into the filter update (the last step). This error term is computed multiple times for any given day index using multiple filters depending on the number of steps for the prediction. For instance, using today's data, denoted by the index n, and the past $M-1$ days, denoted by indices $n-(M-1),\ldots,n-1$, we make a prediction for a future day at index $n+P$, using the present filters. In addition, we use the same filters to make a prediction for day $n+P-1$, using the available data indices $[n-1-(M-1),\ldots,n-1]$, and similarly we continue to make a prediction for every day prior to that up to and including the index $n+1$ (the corresponding data indices for the latter prediction are $[n-(M-1)-(P-1),\ldots,n-(P-1)]$). In other words, every day in the future will have P predictions (P looks) that were made when that point was P days ahead of the index n, $P-1$ days ahead, and so on, until it was only 1 day ahead. So when the index n (i.e. today) turns to $n+1$, i.e. the data for tomorrow becomes available, we have P possible error terms, only one of which can be fed back into the filter update equation. We chose the error term with the smallest magnitude to perform the update.
